# ‘It Got Me Thinking About My Home Environment but Was Also Great Fun’: Using Experiential Methods in Public Involvement Workshops on Indoor Air Pollution in Later Life

**DOI:** 10.1111/hex.70869

**Published:** 2026-09-10

**Authors:** Jennifer Liddle, Gemma F. Spiers, Angela Wearn, Katie H. Thomson

**Affiliations:** ^1^ Population Health Sciences Institute Newcastle upon Tyne UK

## Abstract

**Background:**

Public involvement in health research is increasingly recognised as essential, yet involving older people, particularly care home residents, remains challenging. Creative and arts‐based methods have been proposed as a means of improving accessibility and engagement, but tend to be selected for their perceived general appeal rather than their connection to the research topic. This paper describes an experiential public involvement approach in which activities were deliberately chosen to connect with and embody the sub‐topics of the research.

**Methods:**

Three workshops were held with a group of older people, including care home residents, to inform a research agenda on indoor air pollution. Each workshop employed a hands‐on experiential activity (painting ceramics, attending a glass‐blowing demonstration, and building terrariums); all were aligned with the topic of indoor air quality. Workshops were designed to be informal, enjoyable and accessible, deliberately departing from traditional academic formats.

**Results:**

Ten older people participated, including three care home residents. Workshops generated research priorities and identified important outcomes, including quality of life and dizziness, and acceptable interventions relating to indoor air pollution. Feedback indicated high levels of enjoyment, learning and appetite for continued involvement. The experiential approach was well received, fostering relaxed and meaningful discussion, equal participation between care home residents and community‐dwelling older adults, and engagement that extended beyond the research itself.

**Conclusions:**

Experiential methods, purposefully aligned with research content, offer a distinctive and effective approach to public involvement with older people, including care home residents. This approach extends existing creative and arts‐based methods by embedding a clear conceptual connection to the research topic within activity design, with broader implications for inclusive public involvement practice.

**Public Contribution:**

The workshops described in this paper were themselves the public involvement activity. Older people, including care home residents, actively shaped the research agenda on indoor air pollution by identifying priority outcomes and acceptable interventions. As this was an initial exploration into a new topic area, the research team had no established public contributor contacts with relevant lived experience of or interest in indoor air pollution to involve in co‐designing the workshops. The workshops themselves were therefore the first step in building those relationships, with the intention that members of the group will be involved as genuine partners in any future research. Public contributors have been kept informed of the progress of this work throughout, including during preparation of this manuscript, and it is intended that members of the group will be involved in the development of future funding proposals as co‐applicants.

## 1 Background

Poor air quality is the largest environmental risk to public health in the UK, leading to deteriorating health, long‐term health conditions and early deaths [1, 2]. Public awareness of the negative effects of outdoor air pollution is relatively good [3, 4]. However, the potential impacts of indoor air pollution (e.g. at home) are not as widely understood [5]. Despite a growing population of older people, much of the discussion around improving indoor air quality has not specifically considered what works best for older people. Yet older people are more likely to spend time indoors, and as such theirs are key voices yet to be heard on this topic [6]. As research about indoor air quality for older populations gains traction, it is essential that this is informed by, and focused on, the priorities and experiences of older people.

Public Involvement (PI) is the mechanism through which members of the public and ‘experts by experience’ contribute and inform research. The emphasis is on working ‘with’ or ‘alongside’ members of the public as partners, rather than carrying out research activity ‘to’, ‘about’ or ‘for’ them [7]. The benefits of involving members of the public within research are commonly discussed across the literature, including increasing quality and relevance of research design and processes, more accessible dissemination of research outputs and personal benefits for both researchers and public partners (such as increased confidence and rapport with community members) [8, 9]. Public partners can be involved in a number of ways across the research cycle, including but not limited to the development of research materials and acceptable recruitment processes, contributing to decisions about which outcomes to evaluate in intervention studies or as part of knowledge translation activity with external stakeholders. Recently, many research funders and organisations are increasing efforts for researcher‐public partnerships to begin as early as possible in the research process, to help shape more community‐focused research agendas and contribute to priority‐setting activities [10].

Although public involvement is now widely regarded as an integral component of health and social care research, there can be a number of challenges for both researchers and potential public partners when aiming to establish, work together and sustain an effective collaborative partnership. Research environments can feel intimidating and/or inaccessible to those within traditionally seldom‐heard groups, such as care‐home residents [11]. Past research has also outlined additional challenges in involving older communities within public involvement activity, including limited confidence to contribute, challenges in continuing participation in activities, apathy towards research and communication issues [12, 13].

Accessible PI approaches that incorporate flexibility, respect and value individual abilities are key when aiming to work alongside older groups in a meaningful capacity [13, 14]. Although methods to involve the public in research are well‐established, they continue to evolve in response to the needs of different populations and research contexts. Creative and arts‐based methods are increasingly employed to engage seldom‐heard groups or those otherwise unfamiliar with traditional academic environments [15, 16, 17]. Proponents argue that such approaches foster more meaningful engagement and enhance the accessibility of research for the public. Whilst there is no agreed definition of what constitutes a ‘creative’ method, a recent review suggests they are mainly arts‐based, such as creating animations, writing poetry, sketching, or using mood‐boards [18]. Visual techniques in particular (such as drawing, painting, or observing art and relevant images) are used to encourage communication around health topics and prompt identification of research priorities [19, 20]. Viewing or creating art has also previously been shown to prompt individuals to link their own personal experience with otherwise abstract or hard‐to‐visualise environmental impacts on health [21].

Whilst some past research highlights visual methods as an approach to involving older groups in research (e.g., viewing digital or photographic images to invoke memories and prompt discussion) [22], there remains limited evidence in the literature of their application in involving older people, including care home residents, in research development and priority‐setting activity. Sharing good practice, as well as the outcomes and impact of such activity, is therefore likely to be of value to the research community and contribute to the aims of increasing diverse and inclusive public involvement practice [17, 23].

In our own work on indoor air pollution and older people, we planned to consult with members of the public to help shape and prioritise our research questions. For this, we wished to step away from more traditional forms of engagement and instead use approaches that were engaging and enjoyable for the older people taking part. Not only would a more enjoyable experience help support the longevity of the group, but we posited that it would facilitate meaningful discussion that could inform our research. Importantly, we moved beyond creative methods as typically described in the literature, which tend to be arts‐based in nature, to develop what we describe as *experiential methods*: hands‐on, participatory activities that were deliberately chosen because they connected directly with the sub‐topics of our research. This purposeful alignment between activity and research topic is, we suggest, a distinctive feature of our approach.

This work was initiated by JL, who identified indoor air pollution as an important and underexplored topic for older people and brought together a team of collaborators to begin exploring it. As this was a new topic area for the team, public involvement was considered an essential first step before designing any research questions or studies. The work was funded by a Tilly Hale Award, details of which are provided in the acknowledgements.

In this paper, we describe our approach to engaging with older communities to shape a research agenda on indoor air pollution, detailing the experiential methods used and reporting both the outcomes of this public involvement activity and its impact on our research. Our approach embodies the National Institute for Health and Care Research (NIHR) definition of public involvement as an active partnership between patients, carers and members of the public with researchers that influences and shapes research [7]. In this case, the public involvement activities focused on the exploration and identification of research priorities.

## 2 Methods

The research team comprised early and mid‐career academic researchers with experience in qualitative research, policy, and evidence synthesis related to ageing and older people, but without clinical backgrounds or prior expertise in air pollution. This was a new topic area for all team members. To explore public views on the topic of indoor air pollution, we organised three workshops to be delivered in person. Reporting of this public involvement activity follows the GRIPP2 short form guidance for reporting patient and public involvement in health and social care research [24]. Claude Sonnet 4.6 (Anthropic, 2026) was used as a supplementary aid to support the summarisation of some written content. All outputs were reviewed and verified by the authors.

### 2.1 Recruitment of Public Partners

We advertised this involvement opportunity using various methods, including word of mouth, posters and through the ‘Valuing Our Intellectual Capital and Experience’ (VOICE) online platform for community involvement and engagement [25]. We sought contributors living in the North East with lived experience of health conditions likely to be impacted by air quality, including asthma, chronic obstructive pulmonary disease (COPD) and heart disease. We also endeavoured to recruit people with differing living situations (tenure, building characteristics, number of individuals in household) that can influence air quality due to, for example, the types of heating/cooking fuel used, proximity to traffic, levels of condensation and heating, but also the level of control individuals have over their living environments. We advertised the study using accessible language, including phrases like ‘share your thoughts and experiences to help us design research on household air and health’ and ‘what's important to older people with asthma and other long‐term conditions?’. Our other core target group was care home residents—a population often excluded from mainstream research [12]. Due to ongoing COVID‐19‐related restrictions at the time of recruitment, care home participation was secured exclusively through personal invitations to organisations with whom a member of the team (JL) had an existing professional relationship. We aimed to include around 10 participants at each workshop, with all partaking in all three workshops. Recruitment through VOICE and via the care home meant that individuals self‐selected by expressing interest following our advertisement. We are therefore unable to report how many people were approached but declined to participate.

Our recruitment efforts prioritised diversity in terms of health conditions and housing type, as these were considered most directly relevant to indoor air pollution exposure and vulnerability. Whilst we hoped that recruiting through VOICE, which has a diverse membership, would yield some ethnic diversity within the group, we did not specifically target this, and the group's ethnic diversity was limited as a result. With a limited number of workshop places, budget and time, it was not possible to target more dimensions of diversity, but we recognise that future work should aim to reach a wider range of communities.

### 2.2 Workshops

Workshops were held between December 2022 and March 2023. Our aim was to ensure that care home members were integrated within the group. However, the first workshop was repeated on two occasions so that the activities could be conducted separately in the care home due to ongoing COVID‐19 restrictions. The second and third workshops successfully brought together all members of the group.

The workshops were intentionally designed to break away from a more traditional academic, or formal, format based around documents and paperwork. Instead, each of the three activities was designed to connect directly with a sub‐topic relevant to indoor air quality, and required ‘hands‐on’, sensory or interactive participation—what we describe in this paper as an experiential approach. Activity ideas were initially generated by JL based on the research sub‐topics and a consideration of what might be engaging and accessible for the participant group. Options were discussed with GFS and KHT, and a shortlist of these was then discussed informally with care home staff, before final decisions were made. Each workshop was scheduled for 2 h. Whilst formal ethical approval was not required because this work was classified as public involvement rather than research, the team followed appropriate ethical principles throughout. All data, including paper notes and feedback forms, were stored securely—paper records in locked filing cabinets and digital records on secure university systems. No participant contact details were shared with other participants. All contributors were informed about how information would be collected, stored and used, and informed consent was provided either verbally or via email for the use of photos and quotes included in this paper or any other outputs. Sessions were designed to be relaxed and enjoyable, with refreshments and breaks provided, and no sensitive issues or conflicts arose during any of the workshops. As part of our public involvement method, we sought written and verbal feedback at each workshop to evaluate the success of our approaches and implement any learning in subsequent sessions. Written feedback was collected through a brief questionnaire assessing participant satisfaction and suggestions for improvement, while verbal feedback was elicited through end of workshop reflection and discussion.

Other information was collected from the workshops in the form of notes taken by facilitators and materials produced by participants, for example, the ordering of priority cards and sticky dot voting. These were collated following each workshop to identify key themes and priorities emerging from discussions, which informed the focus of subsequent workshops and, ultimately, our research agenda.

#### 2.2.1 Workshop 1

**Figure 1:**
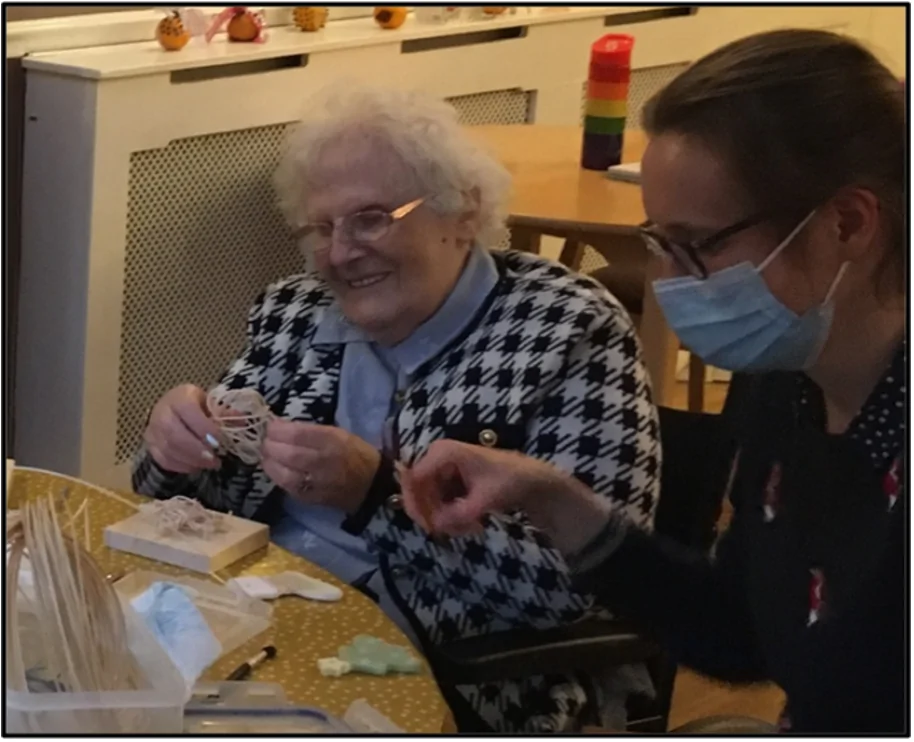
Photograph—painting ceramics in the care home.

**Figure 2:**
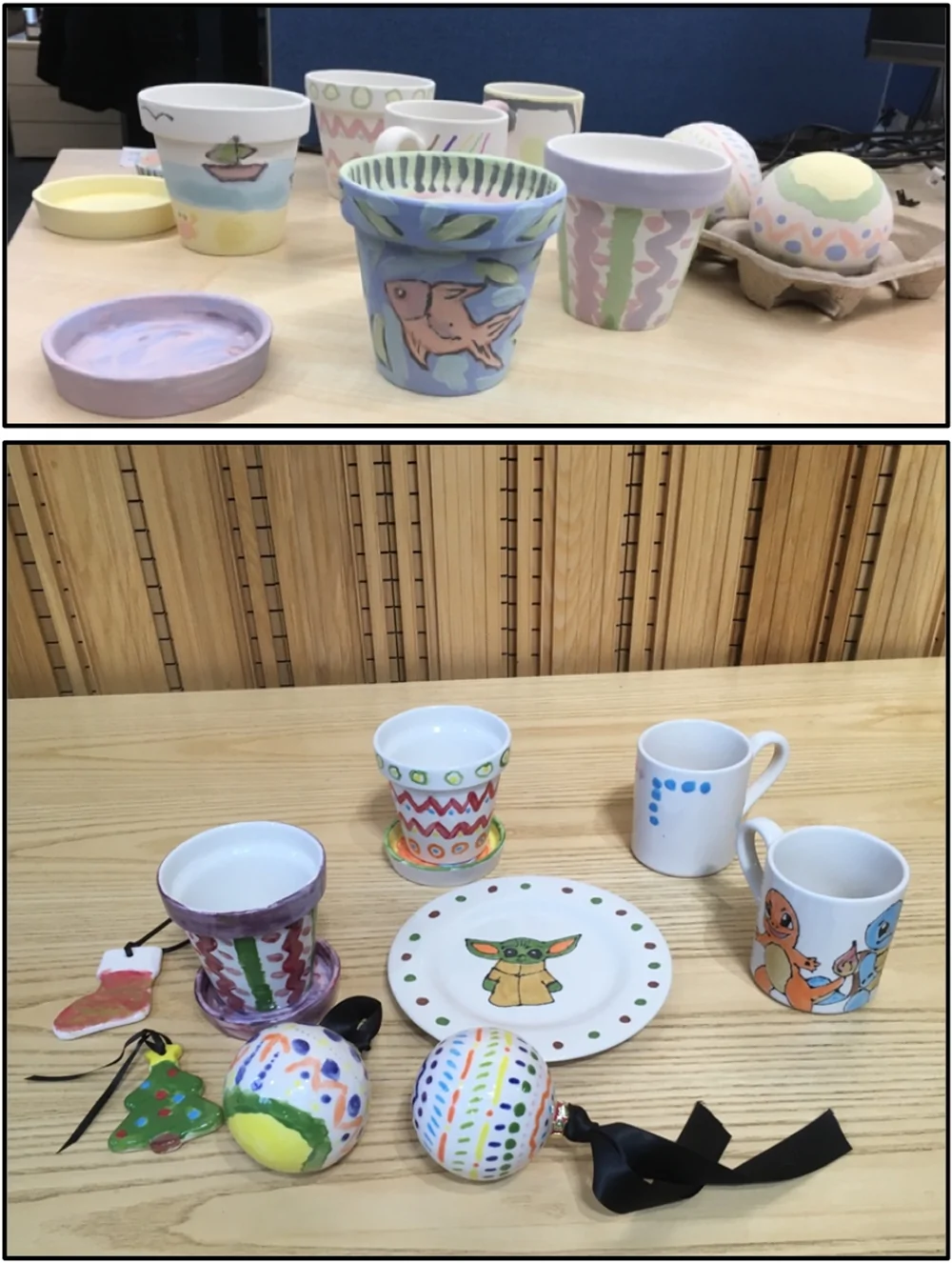
Photographs—painted ceramics before and after firing.

Workshop 1 introduced the topic of indoor air quality, including the household items that can contribute to poor air quality (e.g. emissions from paints and carpets). The activity involved painting ceramics (Figure 1) to connect with thinking about the impact of paint on air quality, which were then fired and returned to participants at the final workshop (Figure 2). The session for care home residents and staff was facilitated by JL and KHT. The session for other group members was facilitated by JL, KHT and GFS. A range of visual illustrations and diagrams of sources of indoor air pollution were included in the table and referenced in discussions. Facilitators gently guided conversations around a range of topics, including participants’ housing, their knowledge and concerns about air quality, any impacts they had noticed on their health and any measures they had taken to improve air quality in their homes.

#### 2.2.2 Workshop 2

**Figure 3:**
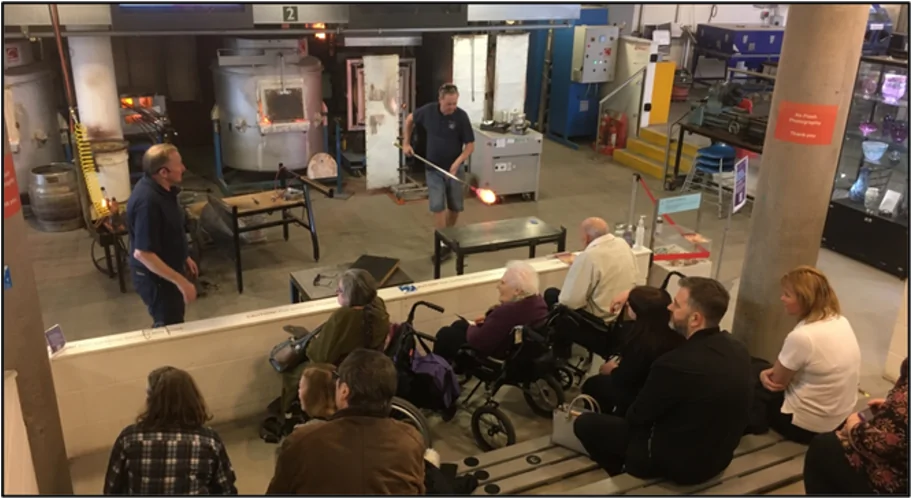
Photograph—glassblowing demonstration.

**Figure 4:**
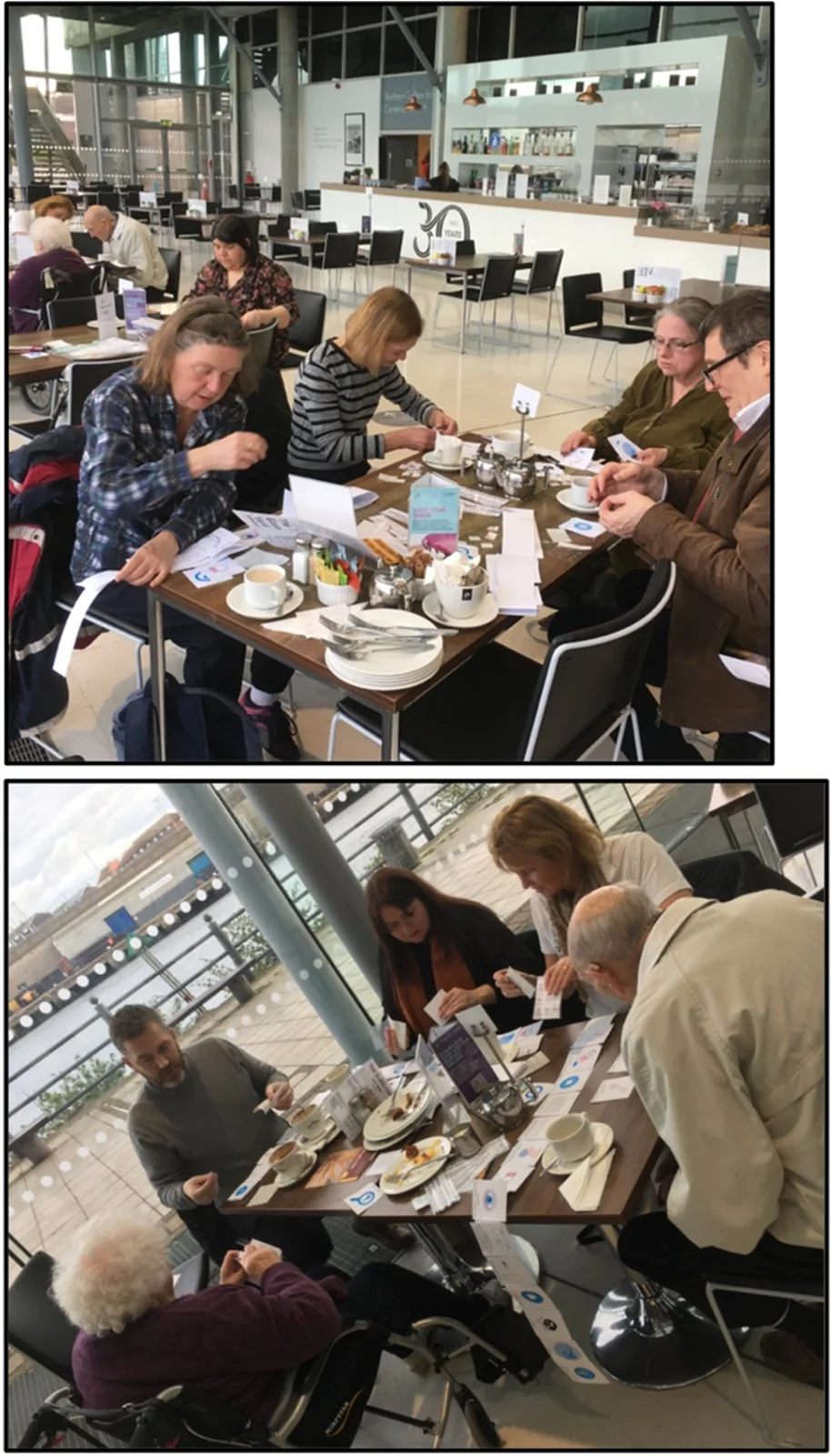
Photographs—prioritising outomes.

Workshop 2 took place at the National Glass Centre. The workshop was facilitated by JL, supported by a senior research administrative colleague, and began with a private glass blowing demonstration in the hot glass studio (Figure 3), followed by time for discussion in the café (Figure 4). The demonstration is linked with the idea of fumes from fires and heating as well as occupational and leisure exposure to pollutants in indoor spaces. The goal of workshop 2 was to identify which health impacts of indoor air quality were important to prioritise in future research. To achieve this, participants were asked to prioritise the health outcomes that would be most meaningful to them when thinking about this topic. Laminated news headlines about the impacts of air quality on health were used to prompt discussion around well‐known and lesser‐known impacts. Participants were given cards illustrating different aspects of health and the potential health impacts of poor air quality. They were asked to rank the perceived importance of these aspects by placing the cards in order along long strips of card, generating interest, laughter and collaborative efforts to physically manoeuvre the items. Participants were also asked to use three stickers to nominate the outcomes of overall most importance to them.

#### 2.2.3 Workshop 3

**Figure 5:**
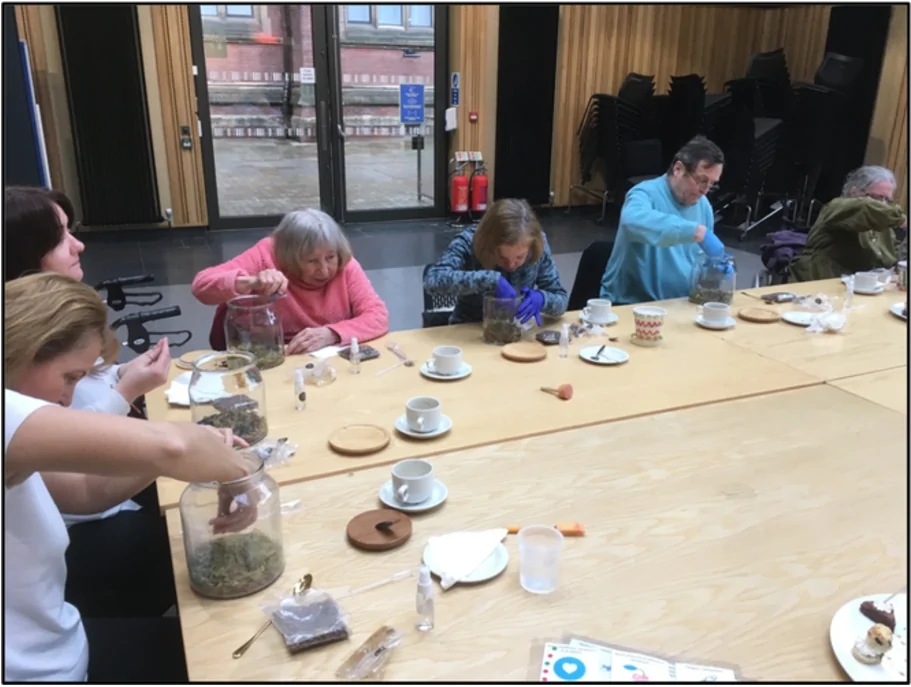
Photograph—terrarium construction.

**Figure 6:**
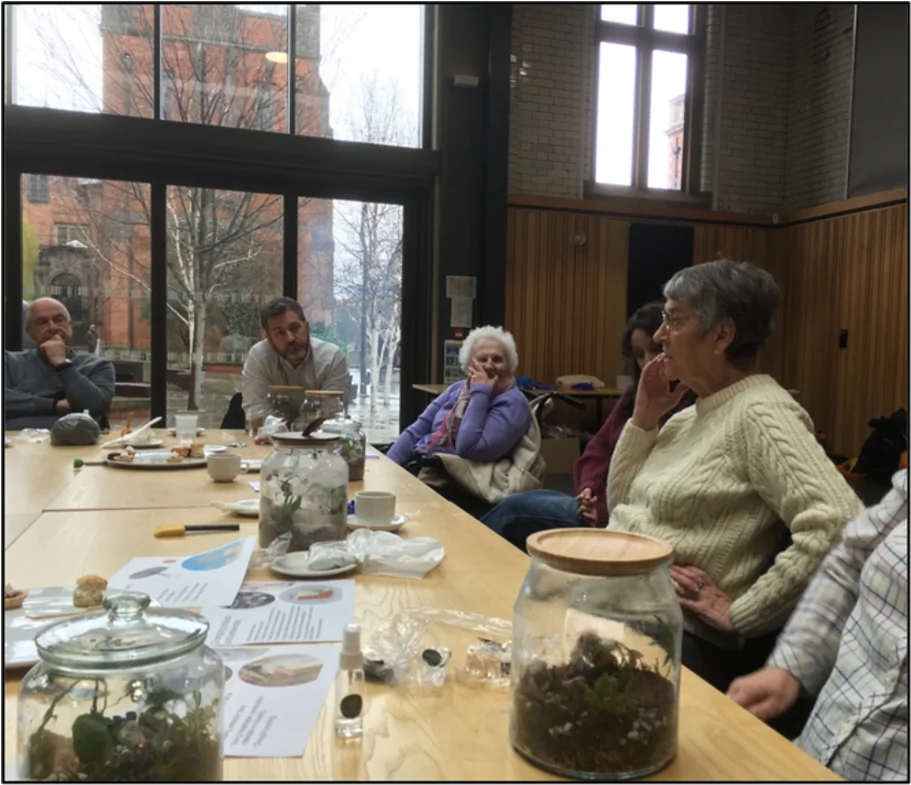
Photograph—group discussion.

Workshop 3 focused on the topic of interventions to improve indoor air quality. The goal of workshop 3 was to identify which interventions to promote indoor air quality may be acceptable to older people and worthy of future research. JL, KHT and GFS facilitated the session. Participants built their own terrariums, linking with the idea of plants having an air purifying role (Figure 5). Visual ‘slides’ of various intervention types were displayed on the tables, and facilitators guided discussion around the feasibility and acceptability of the approaches, which were preferred and why. In the final section of the workshop, participants were asked to reflect on their experiences of being in the group, with the intention of gathering feedback to inform our future public involvement plans and activities (Figure 6).

The aim of the workshop activities was to create a relaxed, enjoyable atmosphere that provided a level of background occupation while discussions continued. In terms of physical accessibility, we ensured that all venues and rooms used were fully wheelchair accessible, which was essential given that the group included three wheelchair users—one community‐dwelling participant who was independently mobile and two care home residents supported by members of care home staff. Refreshments were provided each time, and participants’ travel expenses were reimbursed. Remuneration for attendance and participation was in the form of vouchers, in line with NIHR guidance for public involvement activity [26]. Agendas or preparatory materials were not circulated in advance to preserve an informal, low‐pressure environment for workshop participants. Instead, those participating were informed of the topic of each session, reassured that no preparation was required, and provided with practical details of the venue and timing. Any questions raised in advance were responded to promptly by email. Each participant was also sent a personalised handwritten postcard a few weeks after each workshop, summarising conversations, noting one or two of their individual contributions and outlining outcomes and progress—both as a means of maintaining engagement and as a personal acknowledgement of each participant's contribution.

## 3 Results

### 3.1 Workshop Attendees

We recruited seven older adults living in the community and three care home residents, supported by three care home staff who also contributed in their own capacity. One care home resident was ultimately unable to attend any of the workshops due to personal circumstances. Of the remaining attendees, all attended at least one workshop, with five older adults and two care home staff attending all three. Whilst some participants had previous experience of public involvement in health research, none had previously been involved in research on air pollution.

As this was public involvement work and not research, limited sociodemographic data were collected. However, the group was relatively diverse in terms of sociodemographic characteristics: ages ranged from 65 to 92 (older people) and the group included eight women (two of whom were care home staff) and four men (one of whom was a care home staff member). Housing tenure included own and rented accommodation, including social housing, and housing types included detached, semi‐detached, terraced, bungalow, studio flat and care home. Health conditions represented within the group included asthma, COPD, macular degeneration and high blood pressure. Three individuals were wheelchair users.

### 3.2 Workshop Outcomes

Across the three workshops, our discussions generated outcomes relevant to our research agenda on indoor air quality and older people. In the first workshop, the discussion confirmed the importance of considering ways to improve indoor air quality for older people, and participants conveyed real concerns about indoor air quality alongside the measures they were already taking to address this. Tensions in managing smells in communal care home environments versus the use of air fresheners were discussed, with staff noting they had not previously considered the health impacts of air fresheners.

The second workshop identified outcomes that older people felt were particularly important to address (Box 1), as well as outcomes or ideas they felt were missing from the materials presented, or from research and public debates more generally (Box 2). Outcomes in Boxes 1 and 2 are not presented in ranked order. Participants were asked to place cards in order of importance and to use sticky dot voting to identify their top three priorities; the outcomes listed reflect those that received the most votes across the group, rather than a formally ranked hierarchy. These findings could directly inform outcome selection in future intervention research on indoor air pollution and older people, consistent with evidence that patient involvement in outcome selection leads to inclusion of outcomes that matter most to those affected [27]. Notably, quality of life was emphasised as an important outcome when finding ways to improve indoor air quality, whilst outcomes relating to longevity and mortality were not a primary concern for workshop participants, particularly among those who were care home residents. Dizziness was reported as important in relation to its potential impact on falls and related injuries.

Box 1 **Outcomes identified as most important**

Lung/breathing conditions, symptoms or problems

Heart conditions, symptoms or problems

Dizziness

Cancer

Quality of life

Thinking/cognition/memory problems or symptoms

Viruses/infectious diseases

Nose or throat conditions, symptoms or problems

Blood pressure problems or symptoms

Medications required

Fatigue/exhaustion

Box 2 **Missing outcomes and ideas**

Overall economic impact. & cost of emergency health events, planned care, quality of life, medications and early death

Meaningful and accurate statistics—how monitoring is done, what is measured

Other environmental factors—damp, mould

Research into air quality in public indoor spaces, for example, shopping malls

Other outcomes—diabetes, vomiting

The third workshop generated an in‐depth discussion of interventions, and in particular, which approaches would be acceptable and which participants would be less willing to consider. Interventions promoting behaviour change (e.g. ventilating homes, reducing use of candles) or using products in the home (e.g. air filters) were seen as acceptable where these were practical and inexpensive. There was greater interest in new devices and materials, such as air filters and monitors, though concerns were raised regarding cost, particularly ongoing expenses such as electricity consumption. Structural changes to the home were seen as the least feasible and acceptable, particularly for those renting their accommodation. Policy level changes such as taxation or financial penalties were perceived as less effective, with public education and knowledge provision seen as important to encourage, or accompany, any changes in laws, regulations, taxation or subsidies.

### 3.3 Impact of the Workshops and Feedback

Beyond the specific research insights generated, the workshops also had a broader impact on participants and on our approach to public involvement. As well as informing our understanding of public interest and priorities for research on this topic, the workshop activities were used as opportunities to build longer‐term relationships with public partners to support future research plans. All but one participant agreed to continue their link with this work, with interested members intending to contribute further to the design of research on this topic and be integrated within funding proposals, for example, as citizen expert co‐applicants with associated costings included. The participant who chose not to continue indicated that, although they had enjoyed taking part, they did not have a strong enough interest in the topic to commit to longer‐term involvement.

Feedback ratings were consistently positive across all three workshops, with all responses falling in the top two categories (happy or very happy) and no neutral or negative responses recorded across enjoyment, interest of discussion and satisfaction with the venue (Table 1). Participants also reported high levels of learning and strong interest in ongoing participation (Table 2). Almost all responses indicated ‘yes’ to both questions across all three workshops, with one participant indicating uncertainty about whether they had learnt anything new in the second workshop. All free‐text comments and feedback sent by email were positive (Table 3).

**Table 1:** Workshop satisfaction ratings.

	Very unhappy			Unhappy			Neutral			Happy			Very happy		
Workshop	1	2	3	1	2	3	1	2	3	1	2	3	1	2	3
Did you enjoy the session today?														9	10
Did you find the discussion interesting?											1			7	10
How was the location/venue?												2		8	8

*Note:* Feedback was collected using a visual five‐point smiley face scale to maintain accessibility and consistency with the informal nature of the workshops. Response categories are presented here as text labels.

**Table 2:** Workshop interest ratings.

	No			Maybe			Yes		
Workshop	1	2	3	1	2	3	1	2	3
Have you learnt anything new?					1			7	9
Would you be interested in working with us again to develop a research project on this topic?			1					8	9

*Note:* Feedback was collected using visual tick, question mark and cross symbols. Response categories are presented here as text labels.

**Table 3:** Comments on feedback form.

W3—Any other comments?
Really enjoyed the session and learnt so much!
Great location/venue!
W2—What could we improve next time?
Nothing
It's ok as it is!
Nothing; everything fine
W2—Any other comments?
This was fantastic thank you ☺
Enjoyed the activity and demonstration
Good company, interesting day (glass making and of course our discussion)

The experiential nature of the sessions was particularly well received: participants enjoyed the hands‐on, tactile activities and the opportunity to engage physically alongside discussion. As ongoing, living outputs, the terrariums provided a topic of conversation for ongoing updates between participants and the research team, whilst the painted ceramics served as mementoes of the events. Bringing care home residents and community‐dwelling participants together within the same workshops was a highlight for participants and researchers alike. Care home staff reported that the glass blowing demonstration and visit to the National Glass Centre had been a topic of conversation in the care home for weeks afterwards, and was a particular highlight for one resident who had previously worked in a similar industry. The reach of the workshops extended further still when the care home subsequently produced a video about getting involved in research more generally; notably, many of the activity clips featured in the video were drawn from our workshops in an unsolicited testament to their engaging, unusual and inspiring nature, and to the impact the sessions had made within the care home community.

Participants also appreciated the personal touches that accompanied the workshops, including a handwritten personalised postcard sent to each participant following their attendance. One participant wrote:

It was such a lovely unexpected surprise to get a card thanking me for my participation when really it should be me thanking you for the opportunity. The terrarium is doing well and I find myself praising and encouraging it. And it seems to respond. Keep doing the unexpected.

This individual's subsequent unprompted email feedback illustrates the breadth of impact of the workshops, capturing the value of the experiential activities, the attention to practical detail, and the wider personal significance of participation for some members of the group:

I just want to say thanks to you and the team for organizing such a great session ‐ it got me thinking about my home environment but was also great fun ‐ the time went so quickly. I should have mentioned in my feedback ‘attention to detail’ It crossed my mind in the session, how am I going to carry the terrarium home ‐ but you had thought of that, providing everyone with a canvas carrier. Good thinking ‐ anticipating needs. And another thank you for helping boost my confidence. I don't get out as much since my accident last year and I have been nervous about using taxis independently. My fears were unfounded ‐ everything went safely and smoothly. Thanks for all your help, team ‐ you ensured my safety and comfort. Yet another thankyou for the voucher. I hope you will invite me to any other sessions.

Overall, both the workshop content and the experiential methods employed appeared to be well received by participants, generating meaningful engagement with the research topic alongside high levels of enjoyment and a strong appetite for continued involvement. In contrast to our previous experience using more formal formats without activities, where discussions could often stall and require researcher prompting, the activity‐based approach appeared to facilitate more natural and sustained conversation. Periods of silence that interspersed discussions seemed to reflect natural pauses whilst participants attended to the activity at hand rather than a lack of discussion, and noticeably less prompting was required from researchers to maintain dialogue. Participants shared their views and experiences at their own pace whilst engaging in the workshop activities, contributing to more organic and participant‐led discussions.

## 4 Discussion

This paper describes an approach to public involvement with older people, including care home residents, that sought to move beyond traditional academic formats by employing *experiential methods*—hands‐on, participatory activities deliberately chosen to connect with and embody the sub‐topics of our research. Combining the outcomes of discussions across the three workshops, we were able to identify interventions to prioritise in developing our research agenda on indoor air pollution and to establish which outcomes should be central to the development and evaluation of any such intervention. In doing so, we sustained contact with a group of engaged public partners and achieved consistently positive feedback on our sessions and approach.

A distinctive feature of our approach was the purposeful alignment between activity and research content. Rather than selecting activities primarily for their accessibility or enjoyability, though these were important, each activity was chosen because it related directly to a sub‐topic of indoor air pollution. This distinguishes our use of experiential methods from much of the creative and arts‐based public involvement literature, where activities tend to be selected for their capacity to engage or to lower barriers to participation, rather than for their conceptual connection to the research topic itself. We suggest that this alignment added a layer of meaning to the activities that enriched discussion and helped participants make connections between their everyday lived experience and the research questions under consideration. This is consistent with previous research demonstrating that creative and making activities can prompt individuals to link personal experience with otherwise abstract or hard‐to‐visualise topics [21], but extends this by embedding that connection into the design of the activity from the outset. We recognise, however, that this approach may not be equally applicable across all research topics. The success of experiential methods is likely to depend, at least in part, on the extent to which meaningful activities can be designed that reflect key aspects of the phenomenon under study.

A further consideration in moving beyond the creative and arts‐based methods framing was the risk that such terminology can itself act as a barrier. Arts‐based activities may carry connotations that feel culturally exclusive or unfamiliar to some groups, and the term ‘creative’ can imply a level of artistic skill or confidence that participants may not feel they possess [28, 29]. By contrast, the experiential activities we employed—planting, painting ceramics, visiting a working industrial site—were chosen to feel accessible, practical and enjoyable rather than artistic, and were described to participants in those terms. This approach to framing and activity selection reflects a broader commitment to ensuring that public involvement is genuinely inclusive, rather than accessible only to those already comfortable in academic or arts‐based settings.

A further distinctive aspect of our approach was the decision to hold workshops outside the care home setting, bring care home residents together with community‐dwelling older adults in shared community venues. Public involvement activities with care home residents typically take place within the care home itself or, increasingly, online [17, 30, 31]. The feedback we received suggests that holding workshops in the community contributed significantly to participants’ experience of the session as engaging, enjoyable and out of the ordinary. For care home residents in particular, participation in workshops held in the community offered something beyond the research itself—the opportunity to engage as members of the wider community rather than as residents, on equal terms with other participants and in spaces not defined by their care needs. This would not have been possible without the enthusiasm, practical support and commitment of care home staff, whose role in enabling resident participation was invaluable [17].

The experience of conversing whilst doing was central to the success of the workshops. Previous research has highlighted the particular value of activity‐based discussion for facilitating social interaction, including for men, with practical activities providing a context for conversation without pressure to perform [32, 33]. We found that this dynamic played out well across all participants, with a relaxed and informal atmosphere conducive to discussion of the research focus, and in welcome contrast to more traditional academic workshops with paperwork, flip charts and post‐it notes. The playfulness and physicality of the sessions appeared to lower barriers to participation and foster a sense of equality, not only between researchers and public contributors, but between participants themselves. In a mixed group of community‐dwelling older adults, care home residents and staff, the shared activity created a levelling dynamic in which no participant was positioned as more expert, capable or socially advantaged than another, and in which care home residents participated on equal terms rather than being defined by their care status.

Our approach maps well onto the UK Standards for Public Involvement [34], which provide a framework for good public involvement practice across six domains. In terms of *inclusive opportunities*, we actively sought to include groups not routinely represented in public involvement activity, including care home residents, and designed our approach to be physically and socially accessible, including through the deliberate avoidance of activities or framings that might feel exclusive or unfamiliar to some groups. Our *working together* approach valued all contributions equally and sought to build sustained, mutually respectful relationships, as evidenced by the high levels of continued engagement following the workshops. We invested substantially in *support and learning*, both through the design of accessible and enjoyable activities and through personal communication with participants before, during and after the workshops. The workshops directly addressed the *governance* standard by giving participants a meaningful role in shaping research priorities and intervention focus. Our *communications* prioritised plain language, informality and personal contact, including the handwritten postcards which were specifically appreciated by participants. Finally, the *impact* standard is reflected in our efforts to document and share not only the research outputs generated but the broader value of the public involvement process itself, including its impact on participants’ confidence and wellbeing, in the spirit of identifying and sharing what good public involvement looks like in practice.

In terms of next steps, the immediate priority is to use the priorities and discussions generated in the workshops to inform one or more systematic reviews on indoor air pollution and older people. Beyond this, we intend to work with the group to develop a funding application for a larger research project, in which interested members could contribute as public expert co‐applicants. This larger piece of work would also provide an opportunity to build on the preliminary relationships and learning from this initial work, helping to increase the diversity of public partners involved.

A recurring theme in our reflections on this work is the significant investment of time it requires. Planning engaging and accessible experiential workshops—sourcing and organising materials, arranging accessible transport, communicating personally with participants before and after each session—is considerably more time‐consuming than organising traditional public involvement formats. The personalised postcards, for example, whilst genuinely valued by participants, required substantial time to write thoughtfully for each individual. We were fortunate to receive dedicated public involvement funding to carry out this pre‐project work, without which the experiential approach described here would not have been feasible. Whilst such funding is not always available for priority‐setting or development activity, we would argue that it should be, and that the outcomes achieved here make a strong case for its value. This work, and the positive outcomes it generates, for participants, for the quality of research, and for the reputation of the institutions involved, does not yet feel appropriately recognised within academic structures more broadly. Papers and funding awards remain the primary markers of staff success, and the considerable time required to do public involvement well sits uneasily alongside other pressures and priorities. We would argue that wider systematic change is needed if the aspiration for diverse, meaningful and inclusive public involvement is to be realised in practice. Similarly, whilst the costs of materials and accessible venues and transport are not prohibitive, experiential approaches of this kind are not possible without dedicated resource. In order to keep costs manageable, we sourced and organised materials ourselves. However, this approach requires considerable time, skills, creativity and organisational capacity, which should be appropriately recognised and resourced, alongside standard remuneration costs for participants.

The experience reported here is offered as an example of what is possible when public involvement is approached with intentionality, genuine care for participants’ experience and a willingness to move beyond established formats. The approach has since been recognised as an example of good practice in public involvement with care home residents by the National Institute for Health and Care Research Applied Research Collaboration North East and North Cumbria [35]. We hope it will be of practical value to researchers seeking to involve older people, including those living in care homes, in research development and priority‐setting activity, and will contribute to the growing evidence base for inclusive and innovative public involvement practice.

### 4.1 Strengths and Limitations

A key strength of this work is the deliberate and intentional design of the public involvement approach, in which method and content were consciously aligned rather than selected for convenience or accessibility alone. The use of experiential methods, each connected to a sub‐topic of our research, represents a meaningful departure from both traditional academic public involvement formats and the predominantly arts‐based creative methods described in the existing literature. The positive feedback received, the high levels of continued engagement, and the unsolicited recognition of the workshops by the care home in their own communications about research involvement all speak to the success of this approach in generating meaningful and enjoyable participation. The inclusion of care home residents alongside community‐dwelling older adults, in community settings, was itself a distinctive and successful feature, enabling a breadth of perspective and an equality of participation that a more conventional format would have been unlikely to have achieved.

In terms of limitations, we acknowledge that the group was not fully representative of the diversity of older people in the UK. As this was a public involvement activity rather than research, comprehensive demographic data were not collected, but we note in particular the limited ethnic diversity and lack of representation of care home residents with advanced cognitive decline/dementia [17]. We recognise that further work is needed to develop approaches that are accessible and appealing to a wider range of communities and individuals. Achieving greater diversity in public involvement, particularly with older people and care home residents, remains a challenge for the field more broadly.

As noted in the methods, we took a deliberate decision not to send formal agendas or terms of reference to participants in advance. Whilst this departs from some conventional public involvement guidance, we would not characterise it as a limitation, rather it reflects a considered judgement about what would best serve this particular group, consistent with the principle of tailoring and balancing public involvement approaches to the needs and preferences of those involved to reduce unintended negative consequences [36].

One practical limitation was that the venue used for the first workshop with community‐dwelling participants was uncomfortably cold, due to automated temperature controls in the building that could not be manually adjusted. Whilst this was beyond our control, it serves as a practical reminder that venue comfort—including temperature, acoustics and accessibility—warrants careful consideration and, where possible, advance checking when planning experiential workshops with older people, for whom physical comfort is particularly important to ensuring a positive and productive experience.

Another consideration relating to venue is that holding workshops at external community locations, whilst beneficial for the reasons described, may have presented a barrier to some potential participants with particular mobility or health needs. We note, however, that the decision to hold the second and third workshops outside the care home was made in informal consultation with care home staff. Staff felt that residents likely to be interested in participating would be willing and able to attend. However, this consultation was necessarily limited to residents staff felt might be interested, and may not have captured the preferences or needs of the wider resident population. Ensuring that venue decisions are made with, rather than for, potential participants remains an important principle for future work.

A further reflection concerns the mechanisms for keeping participants informed of the progress and outcomes of the research following the workshops. Maintaining communication with a wider public involvement group over time, beyond a core long‐term team of public partners, presents particular challenges. We have sought to address this through periodic email updates to group members, including about the development of this paper. We hope also to involve the group again in the development of a funding proposal in due course, which would represent meaningful continuation of the involvement relationship.

Finally, as noted in the discussion, the resource‐intensive nature of this approach should be understood in the context of what it achieved, and we would encourage funders and institutions to recognise the value of such investment.

## 5 Conclusion

This paper describes an experiential approach to public involvement with older people, including care home residents, that moves beyond both traditional academic formats and arts‐based creative methods to use hands‐on activities purposefully aligned with the research topic. This approach enabled meaningful engagement with our research agenda on indoor air pollution and generated research priorities that will directly inform our future work.

The approach demonstrates that groups often excluded from public involvement, including care home residents, can participate in ways that are genuinely enjoyable, productive and personally meaningful. Holding workshops in community settings enabled residents to engage beyond their identity as care home residents—an equality of experience that operationalises broader aims of inclusive and person‐centred public involvement practice. The deliberate alignment between activity and research content, and the move away from arts‐based framings that can feel culturally exclusive, are together a distinctive contribution to the public involvement methods literature.

Doing this kind of public involvement well requires time, skill, creativity and dedicated resource, and we would encourage funders and research institutions to recognise and appropriately reward this investment. The outcomes it produces for participants’ confidence and wellbeing, for research quality, and for the goal of diverse and inclusive public involvement demonstrate a strong case for its value. We hope that the experience reported here will be of practical value to researchers planning similar activities with older people, including those living in care homes, and will contribute to a growing evidence base for innovative, equitable and inclusive public involvement practice.

## Author Contributions

**Jennifer Liddle:** conceptualisation, investigation, funding acquisition, writing – original draft, methodology, writing – review and editing, project administration, data curation, supervision, resources. **Gemma F. Spiers:** conceptualisation, investigation, funding acquisition, writing – original draft, methodology, writing – review and editing. **Angela Wearn:** writing – original draft, writing – review and editing. **Katie H. Thomson:** conceptualisation, investigation, funding acquisition, writing – original draft, methodology, writing – review and editing.

## Conflicts of Interest

The authors declare no conflicts of interest.

## Data Availability

The data that support the findings of this study are available on request from the corresponding author. The data are not publicly available due to privacy or ethical restrictions.

## References

[1] Public Health England , Guidance ‐ Health Matters: Air Pollution (Public Health England, 2018).

[2] Royal College of Physicians , Every Breath We Take: The Lifelong Impact of Air Pollution (Royal College of Physicians, 2016).

[3] “Air Quality Information System (AQIS) Review Steering Group , Air Quality Information System Review: Final Report and Recommendations,” (2024), (UK Government, 2024).

[4] Y. Selmani , “A Survey Study Exploring Attitudes, Knowledge and Perceptions of Air Pollution in the United Kingdom,” Public Health Institute Journal no. 6 (2024).

[5] C. Holman , “Why Indoor Air Quality Matters,” Environmental Scientist 30, no. 2 (2021): 2.

[6] M. Bentayeb , M. Simoni , N. Baiz , et al., “Adverse Respiratory Effects of Outdoor Air Pollution in the Elderly,” The International Journal of Tuberculosis and Lung Disease 16, no. 9 (2012): 1149–1161. 22871325 10.5588/ijtld.11.0666

[7] National Institute for Health and Care Research , Briefing Notes for Researchers ‐ Public Involvement in NHS, Health and Social Care, Version 20 (National Institute for Health and Care Research, 2024).

[8] J. Brett , S. Staniszewska , C. Mockford , et al., “Mapping the Impact of Patient and Public Involvement on Health and Social Care Research: A Systematic Review,” Health Expectations 17, no. 5 (2014): 637–650. 22809132 10.1111/j.1369-7625.2012.00795.x PMC5060910

[9] J. Brett , S. Staniszewska , C. Mockford , et al., “A Systematic Review of the Impact of Patient and Public Involvement on Service Users, Researchers and Communities,” Patient ‐ Patient‐Centered Outcomes Research 7, no. 4 (2014): 387–395. 25034612 10.1007/s40271-014-0065-0

[10] C. Grill , “Involving Stakeholders in Research Priority Setting: A Scoping Review,” Research Involvement and Engagement 7, no. 1 (2021): 75. 34715932 10.1186/s40900-021-00318-6 PMC8555197

[11] T. Backhouse , A. Kenkmann , K. Lane , B. Penhale , F. Poland , and A. Killett , “Older Care‐Home Residents as Collaborators or Advisors in Research: A Systematic Review,” Age and Ageing 45, no. 3 (2016): 337–345. 26790454 10.1093/ageing/afv201 PMC4846791

[12] B. Nocivelli , V. Shepherd , K. Hood , C. Wallace , and F. Wood , “Identifying Barriers and Facilitators to the Inclusion of Older Adults Living in UK Care Homes in Research: A Scoping Review,” BMC Geriatrics 23, no. 1 (2023): 446. 37474927 10.1186/s12877-023-04126-3 PMC10360346

[13] I. Schilling and A. Gerhardus , “Methods for Involving Older People in Health Research—A Review of the Literature,” International Journal of Environmental Research and Public Health 14 (2017): 1476, 10.3390/ijerph14121476. 29186055 PMC5750895

[14] T. Burgher , V. Shepherd , and C. Nollett , “Effective Approaches to Public Involvement in Care Home Research: A Systematic Review and Narrative Synthesis,” Research Involvement and Engagement 9, no. 1 (2023): 38. 37268986 10.1186/s40900-023-00453-2 PMC10234794

[15] K. Broomfield , C. Craig , S. Smith , G. Jones , S. Judge , and K. Sage , “Creativity in Public Involvement: Supporting Authentic Collaboration and Inclusive Research With Seldom Heard Voices,” Research Involvement and Engagement 7, no. 1 (2021): 17. 33731228 10.1186/s40900-021-00260-7 PMC7968302

[16] L.‐A. Fenge , “Using Participatory Arts‐Based Approaches to Promote Inclusive Research,” in Handbook of Social Inclusion: Research and Practices in Health and Social Sciences, eds. P. Liamputtong (Springer International Publishing, 2022), 511–526.

[17] M. Davies , L. Irvine , T. Backhouse , et al., “Research Collaboration With Care Home Residents: A Systematic Review of Public Involvement Approaches,” Research Involvement and Engagement 11, no. 1 (2025): 49. 40375340 10.1186/s40900-025-00724-0 PMC12083167

[18] O. R. Phillips , C. Harries , J. Leonardi‐Bee , et al., “What Are the Strengths and Limitations to Utilising Creative Methods in Public and Patient Involvement in Health and Social Care Research? A Qualitative Systematic Review,” Research Involvement and Engagement 10, no. 1 (2024): 48. 38741156 10.1186/s40900-024-00580-4 PMC11092192

[19] M. M. Archibald , S. Makinde , and N. Tongo , “Arts‐Based Approaches to Priority Setting: Current Applications and Future Possibilities,” International Journal of Qualitative Methods 23 (2024): 16094069231223926.

[20] S. Tierney , S. Dawson , A. M. Boylan , et al., “Broadening Diversity Through Creative Involvement to Identify Research Priorities,” Research Involvement and Engagement 7, no. 1 (2021): 3. 33407929 10.1186/s40900-020-00244-z PMC7787225

[21] L. J. Roosen , C. A. Klöckner , and J. K. Swim , “Visual Art as a Way to Communicate Climate Change: A Psychological Perspective on Climate Change–Related Art,” World Art 8, no. 1 (2018): 85–110.

[22] S. Quinton , D. Treveri Gennari , and S. Dibeltulo , “Engaging Older People Through Visual Participatory Research: Insights and Reflections,” Qualitative Research 23, no. 6 (2023): 1647–1668.

[23] C. Imison , M. Kaur , and S. Dawson , Supporting Equity and Tackling Inequality: How Can NIHR Promote Inclusion in Public Partnerships? (NIHR Journals Library, 2022).

[24] S. Staniszewska , J. Brett , I. Simera , et al., “GRIPP2 Reporting Checklists: Tools to Improve Reporting of Patient and Public Involvement in Research,” BMJ 358 (2017): j3453. 28768629 10.1136/bmj.j3453 PMC5539518

[25] “Voice,” accessed April 23, 2026, https://voice-global.org.

[26] National Institute for Health and Care Research , Payment Guidance for Researchers and Professionals Involving People in Research Version (National Institute for Health and Care Research, 2024).

[27] S. Dodd , S. L. Gorst , A. Young , S. W. Lucas , and P. R. Williamson , “Patient Participation Impacts Outcome Domain Selection in Core Outcome Sets for Research: An Updated Systematic Review,” Journal of Clinical Epidemiology 158 (2023): 127–133. 37054902 10.1016/j.jclinepi.2023.03.022

[28] A. Ellis Furman , A. K. Singh , C. Wilson , F. D'Alessandro , and Z. Miller , “‘A Space Where People Get It’: A Methodological Reflection of Arts‐Informed Community‐Based Participatory Research With Nonbinary Youth,” International Journal of Qualitative Methods 18 (2019): 1609406919858530.

[29] D. Fancourt and H. W. Mak , “What Barriers Do People Experience to Engaging in the Arts? Structural Equation Modelling of the Relationship Between Individual Characteristics and Capabilities, Opportunities, and Motivations to Engage,” PLoS One 15, no. 3 (2020): e0230487. 32210465 10.1371/journal.pone.0230487 PMC7094843

[30] L. E. Loria‐Rebolledo , V. Watson , S. Hassan , et al., “Public Contributors’ Preferences for the Organization of Remote Public Involvement Meetings in Health and Social Care: A Discrete Choice Experiment Study,” Health Expectations 26, no. 1 (2023): 146–159. 36335575 10.1111/hex.13641 PMC9854307

[31] K. Micklewright , A. Killett , G. Akdur , et al., “Activity Provider‐Facilitated Patient and Public Involvement With Care Home Residents,” Research Involvement and Engagement 10, no. 1 (2024): 7. 38200589 10.1186/s40900-023-00537-z PMC10782785

[32] C. Milligan , S. Payne , A. Bingley , and Z. Cockshott , “Place and Wellbeing: Shedding Light on Activity Interventions for Older Men,” Ageing and Society 35, no. 1 (2015): 124–149.

[33] B. Golding and A. Foley , “‘How Men Are Worked With’: Gender Roles in Men's Informal Learning,” Paper presented at the 38th Annual SCUTREA… (2008).

[34] UK Public Involvement Standards Development Partnership , UK Standards for Public Involvement (National Institute for Health and Care Research INVOLVE, 2019).

[35] National Institute for Health and Care Research Applied Research Collaboration North East and North Cumbria , Top Tips for Anyone Considering Setting up a Patient and Public Involvement Group in a Care Home (National Institute for Health and Care Research Applied Research Collaboration North East and North Cumbria, 2023).

[36] A. Wearn , K. Brennan‐Tovey , E. A. Adams , et al., “Evaluating Process and Outcomes of Public Involvement in Applied Health and Social Care Research: A Rapid Systematic Review,” Health Expectations 28 (2025): e70160. 39840654 10.1111/hex.70160 PMC11751718

